# Somatic expression of *unc-54* and *vha-6* mRNAs declines but not pan-neuronal *rgef-1* and *unc-119* expression in aging *Caenorhabditis elegans*

**DOI:** 10.1038/srep10692

**Published:** 2015-06-02

**Authors:** Frauke Adamla, Zoya Ignatova

**Affiliations:** 1 Biochemistry, Institute of Biochemistry and Biology, University of Potsdam, Potsdam, Germany; 2 Biochemistry, and Molecular Biology, Dept of Chemistry, University of Hamburg, Hamburg, Germany

## Abstract

Aging is a highly controlled biological process characterized by a progressive deterioration of various cellular activities. One of several hallmarks of aging describes a link to transcriptional alteration, suggesting that it may impact the steady-state mRNA levels. We analyzed the mRNA steady-state levels of polyCAG-encoding transgenes and endogenous genes under the control of well-characterized promoters for intestinal (*vha-6*), muscular (*unc-54, unc-15*) and pan-neuronal (*rgef-1*, *unc-119*) expression in the nematode *Caenorhabditis elegans*. We find that there is not a uniform change in transcriptional profile in aging, but rather a tissue-specific difference in the mRNA levels of these genes. While levels of mRNA in the intestine (*vha-6*) and muscular (*unc-54, unc-15*) cells decline with age, pan-neuronal tissue shows more stable mRNA expression (*rgef-1*, *unc-119*) which even slightly increases with the age of the animals. Our data on the variations in the mRNA abundance from exemplary cases of endogenous and transgenic gene expression contribute to the emerging evidence for tissue-specific variations in the aging process.

Aging affects most organisms and it is characterized by a progressive decline in the molecular activities and physiological integrity over time which decreases the ability to respond to stress, increases susceptibility to disease and finally leads to cellular senescence and death^1^. However, aging is not simply a deterioration of cell activities with time, but is partially controlled by evolutionarily conserved pathways^2^. Tentatively, nine hallmarks are commonly used to describe different levels of aging-dependent alterations in cellular physiology and function, including genomic instability, telomere attrition, epigenetic and transcriptional alterations, loss of protein homeostasis (proteostasis), deregulated nutrient sensing, mitochondrial dysfunction, cellular senescence, stem cell exhaustion, and altered intercellular communication (defined and reviewed in^1^). Importantly, genetic instability is much higher in some tissues, such as postmitotic neurons^3^, implying tissue-specific differences in aging. The time-dependent accumulation of cellular damage resulting from various aging-related physiological alterations is a primary risk factor for developing some human pathologies, including diabetes, cardiovascular disorders, and age-related neurodegenerative diseases^1^,^4^. Furthermore, cellular damage also may significantly enhance and/or modulate the phenotype of mutation-based pathologies, including repeat-expansion neuropathologies, which although caused by inherited mutation exhibit strong age-dependent onset^5^.

Repeat-expansion diseases are linked to a genetic expansion of an unstable amino acid repeat run. In CAG-repeat pathologies, the expansion of CAG stretch encoding polyglutamine (polyQ) over a certain threshold enhances the propensity of the protein to partition in intraneuronal inclusions (for huntingtin protein, implicated in Huntington’s disease, the threshold is approximately 35 glutamines or CAG codons)^6^. Progressive neuronal dysfunction occurs far earlier than the formation of discernible aggregates^7^ and emerging evidence suggests that not only CAG repeat-dependent deterioration but also age-dependent cellular damage contribute to the disease phenotype^5^. Although the length of the CAG run correlates with the age of onset and disease severity, substantial variability in onset among individuals with the same repeat length implies that additional cellular and environmental factors play modulating roles^8^. Moreover, the aggregation of pathological polyQ proteins correlates with alterations in the nutrient sensing integrity in yeast model^9^, causes transcriptional changes in mouse model^10^, dysregulates energy homeostasis and mitochondrial function in mammalian cells^11^, and compromises regulation of proteostasis in *Caenorhabditis elegans*^12^,^13^. Taken together, these comprise at least four of the categories that describe cellular aging^1^. Clearly, CAG-repeat pathology and aging are intertwined. Yet, it is unclear if polyQ mRNA is steadily expressed with time and the progressive disruption of cellular activities results from accumulated misfolded, aggregation-prone protein^14^.

*C. elegans* development is accompanied by substantial quantitative changes in gene expression^15^. Compared to development, aging appears to be associated with fewer alterations in gene expression pattern^16^,^17^, although large sets of genes participating in many processes that are fundamental to homeostasis and energy metabolism are significantly affected during aging in human, mice and *C. elegans*^18^,^19^. Several genes show consistent changes with aging in many tissues^20^ despite their different rates of aging^21^. Ribosomal RNA exhibits age-dependent stability^22^ while the steady-state level of single protein-coding transcripts varies as a function of the chronological age, with some mRNAs decreasing and others increasing in the time course of aging^17^ However, it remains unclear whether tissue-specific changes in endogenous mRNA expression during aging reflect the behavior of transgenic transcripts controlled under the same tissue-specific promoters. Here, we analyzed transgenic animals expressing polyQ-variants with pathological and non-pathological length under the control of well-characterized muscular (*unc-54*), intestinal (*vha-6*) and pan-neuronal (*rgef-1*) promoters. By early adulthood, polyCAG-mRNA decreased in both intestine and muscle tissues independent of the CAG-repeat length. Intriguingly, we also monitored aging-dependent alterations in the steady-state mRNA levels of endogenous transcripts whose expression is under the same promoters as the transgenes and observed decline of endogenous *unc-54, unc-15* or *vha-6* mRNA in the transgenic, polyQ-expressing nematodes and also in the wild-type *C. elegans* strain. In contrast, the polyCAG-mRNA in pan-neuronal tissues slightly increased with aging. The mRNA of pan-neuronal marker gene showed the same trend: it increased with aging in both wild-type and polyQ-expressing transgenic nematodes. Furthermore, analysis of transcriptome-wide expression profiling in *C. elegans*^18^ corroborates our observations with single transcripts in muscles and pan-neuronal tissues. Together, these results show tissue-specific differences of the mRNA steady-state level during aging.

## Results

### Transgenic polyCAG mRNA decreases with age in intestine and muscle tissues

**Figure 1:**
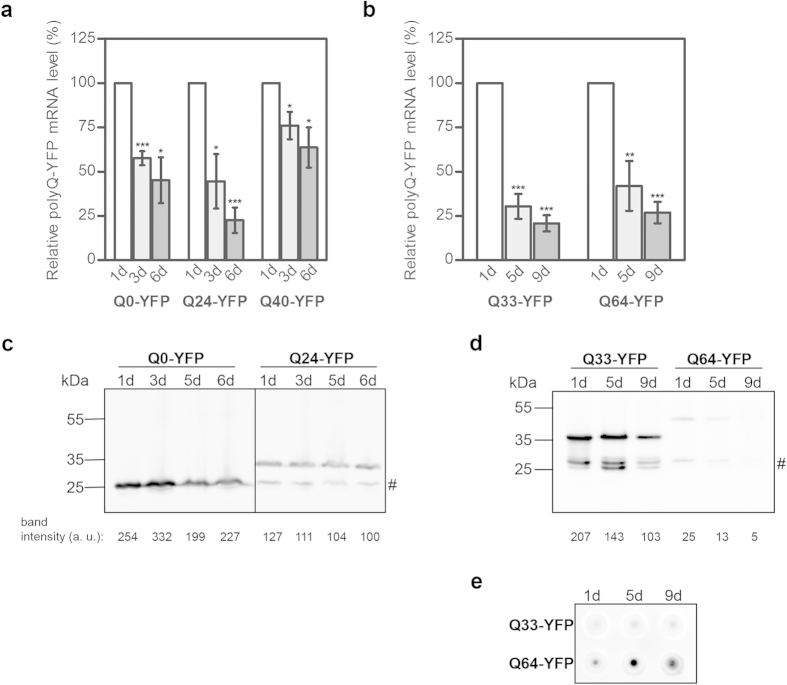
The expression of polyCAG transgene decreases in aged *C. elegans*. qRT-PCR quantification of polyCAG-mRNA expressed in muscle (**a**) and intestine (**b**). The mRNA expression levels were normalized with *act-2* as a reference housekeeping gene. Data are presented as relative values normalized to the first day of adulthood ± SEM (n = 3-4).*p < 0.05, **p < 0.01, ***p < 0.001. Representative immunoblots of the soluble monomeric polyQ-YFP variants expressed in muscle cells (n = 2) (**c**) and intestine (n = 3). (**d**) immunostained with GFP-antibodies. 50 animals expressing the muscle Q0-YFP und Q24-YFP, 30 for the intestine Q33-YFP or 60 for the Q64-YFP were analyzed. # denotes the YFP band of the Q0-YFP expressing animals; in the other *C. elegans* strains this band may have arisen from alternative initiation since the Met-encoding AUG codon is preserved at the start of the YFP sequence. The numbers under the blot represent the relative intensity of the corresponding Q-YFP band. Note that Q64-YFP aggregates and decreases the amount on soluble monomer protein. (**e**) Representative filter-retardation immunoblot of nematodes expressing Q33-YFP (50 animals) and Q64-YFP (50 animals) immunostained with GFP-antibody. Note that Q33-YFP does not form detergent-resistant aggregates.

To assess changes in the level of mRNA transgenic to the nematode genome, we analyzed transgenic *C. elegans* animals expressing polyCAG mRNA in various tissues. The CAG-repeat expansion modulates the aggregation propensity of the host protein in a manner that mirrors the disease threshold; proteins with CAG repeats in the non-pathological length (<35 CAG) are expressed mostly in a soluble form, while expansion over 35 CAG increases the aggregation propensity of the host protein in a length-dependent fashion^6^. We first analyzed transgenic *C. elegans* that express in muscle tissues different Q-YFP fusions with non-pathological (Q0, Q24) and pathological (Q40) repeat lengths whose aggregation behavior resemble the aggregation behavior of huntingtin (htt) protein in Huntigton’s disease (HD)^23^. The transgene is chromosomally integrated and expressed under the *unc-54* promoter in the muscle tissues^23^. Nematodes at different stages of adulthood were collected at one, three and six days. The total RNA was isolated and subjected to qRT-PCR using YFP/CFP-specific primers (Supplementary Table S1). For all three strains, we observed a progressive decrease in the polyCAG-mRNA levels after the first day of adulthood (Fig. 1a).

This raised the question as to whether this pattern is muscle-specific. Thus, we also analyzed strains expressing polyQ-YFP fusions with pathological and non-pathological Q-repeat length expressed in the intestine under the control of the *vha-6* promoter. Similar to the muscle-expressing polyQ-YFP animals, the intestine-expressing nematodes resemble the length-dependent polyQ aggregation phenotype: discernible aggregates are formed early in the time course expression of the pathological Q-YFP variant (Q64-YFP) but not when expressing the non-pathological Q33-YFP protein^24^. We used the same primer set to quantify the polyCAG-mRNA and observed a progressive decrease in the mRNA steady-state levels for both non-pathological (Q33-YFP) and pathological (Q64-YFP) with the chronological age of the nematodes (Fig. 1b). The drop in the mRNA level is independent of the CAG-repeat length as revealed by two-way ANOVA. Notably, for nematodes expressing Q-YFP proteins in intestine, the mRNA level did not decrease gradually with age but rather dropped significantly after the first day of adulthood and then decreased with smaller increments (Fig. 1b). In all experiments, mRNA expression levels were normalized using *actin-2* (*act-2*) as a reference housekeeping gene (Fig. 1a,b). The changes of the polyCAG-mRNA levels were independent of the annealing position of the primer set within *act-2* gene (Supplementary Fig. S1a) and showed the same pattern when another housekeeping gene was used for normalization (Supplementary Fig. S1c).

The protein level of the most variants with a Q-length in the non-pathological range (Q24-YFP, Q33-YFP) also decreased throughout the time-course of analysis for muscle and intestinal expression (Fig. 1c, d). However, the decline in protein amount was modest compared to the polyCAG mRNA decrease during adulthood (compare Fig. 1a, c or Fig 1b, d). The amount of the soluble Q64-YFP monomer also decreased with the chronological age of nematodes (Fig. 1d), but is difficult to interpret because Q64-YFP formed SDS-insoluble aggregates in the time course of expression (Fig. 1e). Detergent-resistant or SDS-insoluble aggregates are indicative of formation of prefibrillar or fibrillar aggregates^25^ which cannot be quantitatively resolved by SDS gel electrophoresis and were retained by filtration on acetate membranes^26^ (Fig. 1e).

### Endogenous mRNA also declines in muscle and intestine with aging

**Figure 2:**
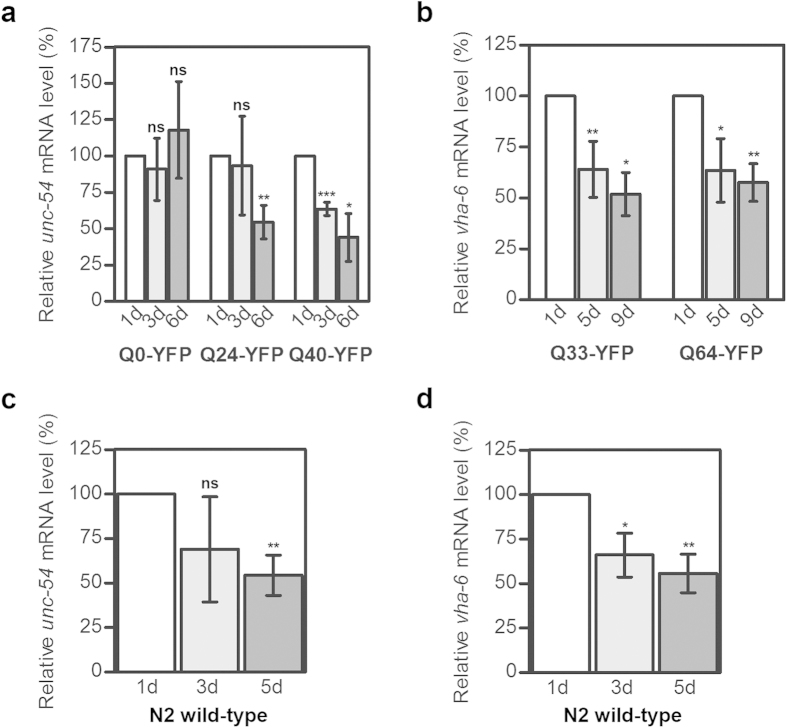
The expression of the tissue-specific genes decreases with age. The mRNA level of *unc-54* (**a**, **c**) and *vha-6* (**b**, **d**) measured by qRT-PCR at different stages of adulthood in transgenic animals expressing Q-YFP variants (**a**, **b**) and in N2 wild-type nematodes (**c**, **d**). *act-2* was used as a reference housekeeping gene for normalization. Data are presented as relative values normalized to the first day of adulthood ± SEM (n = 3-4).*p < 0.05, **p < 0.01, ***p < 0.001, ns, non-significant.

The observation of age-related reduction in the polyCAG mRNA expression levels of transgenic Q-YFP in two different tissues raises the question as to whether this effect is also a feature of the endogenous mRNA. We next measured the expression level of nematode-specific genes, myosin heavy-chain (*unc-54*) and vacuolar H-ATPase (*vha-6*), whose promoters also control the expression of Q-YFP constructs in muscle or intestine, respectively. To compare to the expression of the mRNA of the Q-YFP variants, we used animals of the same age: at one, three and six days of adulthood for the *unc-54* expression and at one, five and nine days of adulthood for *vha-6* expression. In nematodes expressing Q24-YFP and Q40-YFP, *unc-54* mRNA decreased with age, while in Q0-YFP expressing strains it remained constant (Fig. 2a). Similarly, in the intestine the *vha-6* mRNA level decreased in Q33-YFP and Q64-YFP expressing nematodes (Fig. 2b). The *vha-6* mRNA decrease in animals expressing the Q-YFP transgene was independent of the primer set used in the qRT-PCR (Supplementary Fig. S1b, Supplementary Table S1).

These data suggest that the amount of mRNA for nematode-specific genes expressed in muscle tissues or intestine under the control of the same promoter as the Q-YFP transgene also decreased during aging of the transgenic *C. elegans*, resembling the trend observed for the transgenic polyCAG-YFP mRNA. Since the level of *unc-54* mRNA in the muscular cells of Q0-YFP expressing nematodes was not affected, we tested whether there is a Q-length dependent effect on the stability of the nematode-specific mRNA level. Thus, we analyzed the mRNA expression levels of *unc-54* in muscular cells and *vha-6* in intestine of the wild-type N2 strain. Notably, the levels of *unc-54* and *vha-6* mRNA decreased with age in N2 nematodes (Fig. 2c, d). Similar to the transgene Q-YFP-expressing *C. elegans*, a significant reduction of *unc-54* mRNA expression occurred later in the adulthood, at day 5, while the decrease in the first days was insignificant (compare Fig. 2a,c). The same pattern of *unc-54* mRNA reduction was also observed with another primer set (Supplementary Fig. S2a). Furthermore, we observed a similar trend of mRNA decline in N2 wild-type *C. elegans* with another muscle-specific gene *unc-15* that encodes a paramyosin ortholog (Supplementary Fig. S2b). In the intestine, the decrease in the *vha-6* mRNA in the N2 strain resembled the pattern of *vha-6* mRNA in Q-YFP expressing animals and declined significantly from day three on (compare Fig. 2b, d). In sum, the mRNA of nematode-specific *unc-15, unc-54* and *vha-6* genes display similar decline in muscle and intestine over aging as observed in transgenic animals expressing different Q-YFP variants.

### PolyCAG transgene- and tissue-specific-mRNA increases in pan-neuronal tissues

**Figure 3:**
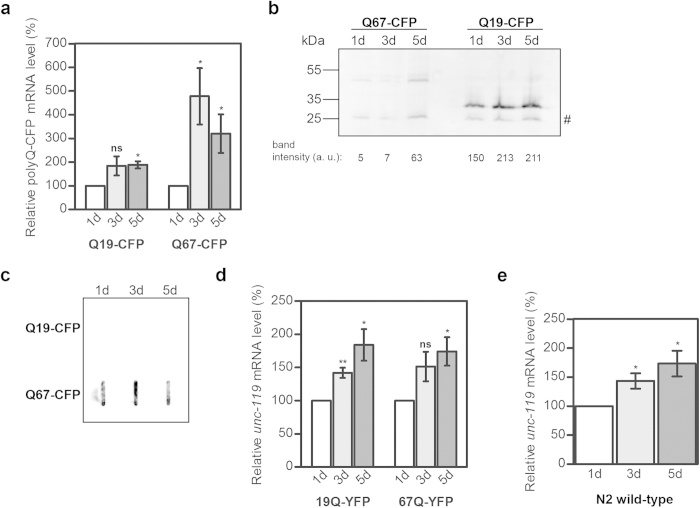
The expression of polyCAG transgene and tissue-specific gene increases with age in pan-neuronal cells. Quantification of the pan-neuronal expression of transgenic polyCAG-mRNA (**a**) and cell-specific *unc-119* mRNA (**d**, **e**) in aged transgenic animals expressing Q-CFP variants (**a**, **d**) and in N2 wild-type animals (**e**) measured by qRT-PCR. *act-2* was used as a reference housekeeping gene for normalization. Data are presented as relative values normalized to the first day of adulthood ± SEM (n = 3-5). *p < 0.05, ** *p* < 0.01, ns, non-significant. (**b**) Representative immunoblot (n = 3) of lysates from 150 animals with pan-neuronal expression of Q-CFP visualized with GFP-antibodies. The numbers under the blot represent the relative intensity of the corresponding Q-CFP band. (**c**) Filter-retardation immunoblot of *C. elegans* expressing Q19-CFP (150 nematodes) and Q67-CFP (150 nematodes) immunostained with GFP-antibody. Note that Q19-CFP does not form SDS-insoluble aggregates and was not retained on the membrane.

Neurons are the primary cellular target of neurological disorders. Thus, we also measured the steady state mRNA amount of Q-CFP variants with CAG-repeat length in the non-pathological (Q19) and pathological (Q67) range in neuronal tissues. The transgene is chromosomally integrated and expressed under the neuron-specific promoter of the *rgef-1* gene^27^. These strains express polyQ-CFP fusion proteins throughout the nervous system, termed pan-neuronal, and show length- and age-dependent aggregation and toxicity for CAG repeats^27^ similar to the Q-length dependent phenotype described in *C. elegans* expressing polyQ proteins in the muscle and intestine tissues^23^,^24^. We collected nematodes at one, three and five days of adulthood and quantified the polyCAG mRNA by qRT-PCR (Supplementary Table S1). Unlike the muscular and intestinal polyCAG-mRNA expression, the neuronal polyCAG mRNA increased with age in both Q19-CFP and Q67-CFP nematodes (Fig. 3a). While the Q19-CFP-expressing animals showed a steady increase of mRNA, the Q67-CFP nematodes exhibited much greater increase but with larger fluctuations in the mRNA levels between day 3 and day 5 (Fig. 3a). The protein amount for both Q19-CFP and Q67-CFP also increased during the adulthood (Fig. 3b). Note that Q67-CFP expressing animals form SDS-insoluble aggregates (Fig. 3c) and thus their expression, similar to the nematodes with intestinal expression of Q-YFP variant in the pathological threshold (Fig. 1d,e), cannot be quantitatively resolved on SDS-PAGE.

The pan-neuronal expression of polyQ-CFP constructs is under the control of the neuron-specific promoter of the *rgef-1* gene that encodes Ras nucleotide exchange factor. Any attempt, however, to analyze its mRNA expression in N2 nematodes failed, most likely due to its inherently low expression which is under the detection limit of the qRT-PCR approach. Therefore, we chose another gene, *unc-119*, which is often used as a pan-neuronal marker^28^. Intriguingly, for both Q-CFP expressing transgenic animals and N2 wild-type nematodes, an increase in mRNA levels (Fig. 3d, e) resembled the changes of the polyCAG-mRNA levels (Fig. 3a). In contrast to the fluctuations observed for the Q67-CFP mRNA (Fig. 3a), *unc-119* steadily increased with age with somewhat similar increment (Fig. 3e). Given that microarray data in mouse can sometimes show unstable expression of β-actin^29^, we considered normalization using another house-keeping gene, *ama-1* (Supplementary Fig. S2c) and we observed the same trend of mRNA increase as for *act-2*-normalized expression (Fig. 3e). Together, our data show that both tissue-specific (*unc-119*) and transgene mRNA controlled under the *rgef-1* promoter increase with age in pan-neuronal tissues in wild-type and transgenic animals.

### Global tissue-specific changes of mRNA expression during chronological age

**Figure 4:**
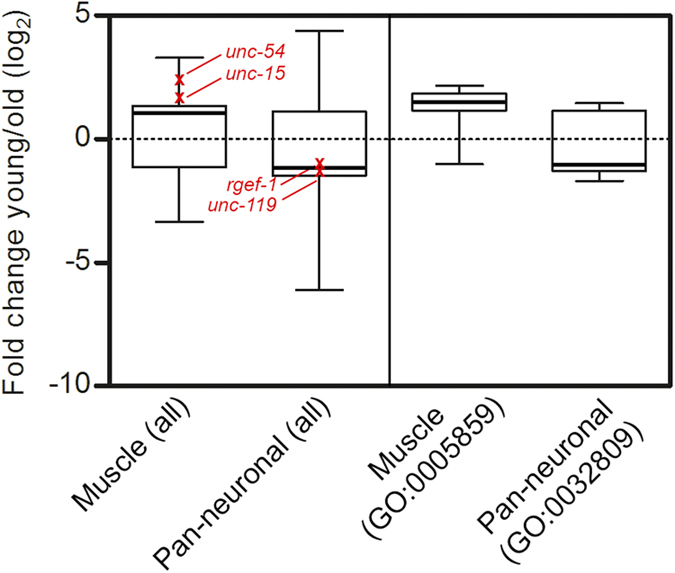
Global tissue-specific alterations of transcripts with age in muscle and pan-neuronal cells. Box-plot analysis of all significantly changed transcripts in muscle and pan-neuronal tissue (all, left side of the plot) with the chronological age from the microarray data^18^. The thick horizontal line in the box plots represents the median. The changes detected in the microarray data^18^ for *unc-54* and *unc-15* in muscle and *rgef-1* and *unc-119* in pan-neuronal cells are highlighted in red. GO:0005859, muscle myosin complex; GO: 0032809, neuronal cell body.

The observed tissue-specific variations in the steady-state level of tissue-specific transcripts (i.e. the decline of intestinal *vha-6* and muscular *unc-54* and *unc-15* and the slight increase in pan-neuronal *unc-119* and *rgef1* transcripts), prompted us to address whether this is a specific feature of these particular transcripts. We analyzed the global tissue-specific changes in the mRNA expression pattern in the aging time-course of previously published microarray data^18^. In muscle, the majority of the transcripts that significantly changed during aging undergo a decline in the mRNA amount (see the median value for ‘muscle all’, Fig. 4). In contrast, in pan-neuronal tissues the larger fraction of the transcripts increases with aging (see the median value for ‘pan-neuronal all’, Fig. 4). In both sets, however, a sizeable set of transcripts shows the opposite trend with chronological age, i.e. increased amounts in muscle and decreased in pan-neuronal tissues (bottom whisker for muscles and upper whisker for pan-neuronal in Fig. 4). We also analyzed the Gene Ontology (GO) term^30^ comprising the genes of interests, muscular *unc-15* and *unc-54* and pan-neuronal *unc-119* and *rgef-1*. Globally, the two selected groups resembled the expression pattern observed for all genes (Fig. 4). Thus, the microarrays corroborated our observations (Fig. 4, right side of the plot). Similar to our observation, the microarray analyses detect a significant aging-dependent decrease of the *vha-6* transcript^18^. For intestine, however, we could not perform any global analysis because of the small set of intestine-specific genes in the data set. In part it might be a reflection of incomplete annotations in *C. elegans* GO terms. Together, these analyses show that transcripts in muscle tend to decline with chronological age while in pan-neuronal tissues the mRNA level tends to increase. However, the fact that sizeable sets of transcripts in both muscle and pan-neuronal tissues oppose these trends suggests variability in the expression signatures, as observed in human muscles^31^.

## Discussion

We show tissue-specific and age-dependent differences in the mRNA expression levels of some chromosomally encoded genes and transgenes (polyQ-YFP/CFP variants) expressed under the control of well-characterized muscular (*unc-54*), intestinal (*vha-6*) and pan-neuronal (*rgef-1*, *unc-119*) promoters in *C. elegans*. While the mRNA level of nematode-specific *unc-54* and *vha-6* transcripts and that of the transgene (polyQ-YFP) controlled by the same promoters declines with age, the mRNA level under the pan-neuronal *rgef-1* or *unc-119* promoters is more stable and even increases with the age of the animals. Transcriptional profiling in young (one-day) and adult (six-day) animals corroborates these observations^18^: the steady-state levels of the majority of mRNAs significantly deregulated in aging are reduced while in pan-neuronal cells they increase (Fig. 4). Similarly, in agreement with our observation of *unc-54-*dependent deterioration of myosin expression, a trend towards reduced type 2a to type 1 myosin heavy chain in microarrays from muscles of aged human skeletal muscle is observed^32^. However, promoter instability, often associated with aging^33^, cannot explain the observed effects: muscular *unc-54* or intestinal *vha-6* promoters show stable postembryonic expression from larvae stage throughout adulthood^34^,^35^. Various *C. elegans* tissues deteriorate differently^36^. Age-associated alterations in myosin^37^ or loss of intestinal microvilli and nuclei^38^ are considered to be signs of muscular and intestinal deterioration, respectively, but sensory and motor neurons do not show obvious signs of neuronal degeneration by advanced *C. elegans* age^36^. In light of these observations, the stability of the transgenic polyCAG mRNA or tissue-specific mRNA in pan-neuronal cells (Fig. 4) could be a result of variations in the aging rate in different tissues^21^, with neurons preserving higher integrity with age compared to that of muscle and intestine.

The steady state mRNA level is determined by the mRNA synthesis (transcription) and decay rates. Alternatively, tissue-specific differences in mRNA degradation can also contribute to variations in the mRNA levels among tissues^39^ through differential expression of the components of the mRNA decay machinery, e.g. ribonucleases and/or associated factors^40^. Interestingly, the expression levels of various proteins known to promote mRNA degradation are lower in cells differentiated to neutrons with retinoic acid^29^; these proteins, at least in part, may decrease the mRNA decay rate and contribute to enhanced mRNA level in pan-neuronal tissues. Thus higher mRNA levels of some mRNAs can also be an indirect result of accumulated cell damage during aging. Supportive for this are global measurements of mRNA levels in yeast in response to environmental stress which show a non-monotonic dependence between mRNA synthesis and decay rate which differently modulates the mRNA steady state level^41^.

In all three tissues, the alterations of the polyQ protein levels, albeit modest as compared to the mRNA changes, mirror the transgenic polyCAG mRNA changes. The differences in the magnitude of decreases in protein and mRNA levels might be explained with the different half-lives of proteins and mRNA^42^,^43^ or by aging-induced collapse of the proteostasis network, i.e. the imbalance between protein synthesis, folding and degradation^4^. Supportive for the latter is the age-related loss of proteostasis which strongly influences polyQ aggregation and polyQ protein synthesis^13^,^44^,^45^.

In sum, our results show variations in the amount of transgenic polyCAG mRNA and some tissue-specific mRNAs during the aging in intestine, muscle and pan-neuronal cells in *C. elegans.* In part, this trend also applies for other transcripts^18^, although it is not a global response of all mRNAs (Fig. 4). Besides variations among the tissues, expression patterns during aging also differ between the same cell types: in primates and mouse, cerebellar neurons have different pattern of aging than cortical neurons^46^,^47^. Furthermore, the effect on cellular physiology may vary depending on the expression level of a gene. Usually, even a modest change in the abundance of low-abundance transcripts produces dramatic changes in gene expression, while for abundant transcripts the small alterations in mRNA level have little effect^48^. In particular, the CAG repeat proteins implicated in various diseases are usually expressed at very low levels under basal conditions; quantitative measurements in the primary expression tissues are thus missing. Of particular importance, even a small age-dependent mRNA increase, as we detected for the polyCAG model mRNA in pan-neuronal tissues, may in general enhance the expression of the disease protein over its basal level. In this context, our findings suggest that together with the aging-induced alterations in cellular physiology^1^, aggregation-prone disease protein may risk enhanced accumulation because of age-related deregulation of its steady-state mRNA level.

## Methods

### *C. elegans* strains and maintenance

The following *C. elegans* strains were used: wild-type strains, Bristol N2; strains expressing CAG repeat transgene in muscle with non-pathological AM134 [*rmIs126 [unc-54p::Q0::YFP*], AM138 *rmIs130 [unc-54p::Q24::YFP]* or pathological CAG length AM141 *rmIs133 [unc-54p::Q40::YFP]*; strains expressing transgenic CAG mRNA in neurons with pathological AM44 *rmIs190 [F25B3.3p::Q67::CFP]* or non-pathological CAG length AM49 rmIs172 *[F25B3.3p::Q19::CFP]*; strains expressing CAG repeat transgene in intestine with non-pathological GF72 *dgEx72 [pAMS54 vha-6p::Q33::YFP + rol-6(su1006)* + pBluescript II] or pathological CAG length *GF83 dgEx83 [pAMS56 vha-6p::Q64::YFP + rol-6(su1006)* + pBluescript II]. In the strains AM134, AM138, AM141, AM44 and AM 49 the transgene is stably chromosomally integrated, while GF72 and GF83 strains express the transgene extrachromosomally. AM134 was a kind gift from Janine Kirstein-Miles (FMP, Berlin), all other strains were obtained from the *Caenorhabditis Genetics Center*.

Strains were maintained at 20 °C for the experiments and *C. elegans* handling was performed using standard methods^49^. Briefly, nematode populations were synchronized using hypochlorite bleaching and then cultured on NGM plates seeded with *Escherichia coli* OP50. During the reproductive stage, animals were moved to fresh NGM plates every second day to keep the set of animals at uniform age and avoid interference of the offspring.

### RNA isolation and RT-PCR

Total RNA was isolated from the synchronized *C. elegans* populations of different time-points during adulthood using TRI Reagent (Sigma). 100 to 200 synchronized animals were collected into a drop of nuclease free H_2_O, then 500 μl of TRI Reagent was added and samples were frozen and thawed in three repeating cycles to break the animals cuticles. Further extraction of total RNA followed the manufacturer’s protocol (Sigma). The integrity of the RNA was analyzed either using 1% formaldehyde agarose gel electrophoresis or with RNA 6000 Nano Chip (2100 Bioanalyzer; Agilent Technologies). Thereafter, 0.5 μg of isolated RNA were treated with RNase-free DNase I (Thermo Scientific) and used for cDNA synthesis with Oligo(dT)-primers and Revert Aid Reverse Transcriptase (Thermo Scientific). The cDNA in 1:3 dilution was used in a quantitative real-time PCR (qRT-PCR) (SYBR green-based approach, Qiagen). Each reaction was performed in duplicates and each run included control samples containing either no template or no reverse transcribed transcript. A detailed list of the primers is included in SI Material and Methods. Samples were run and analyzed with MxPro QPCR software (Agilent Technologies) and mRNA quantification was done by using the 2^-ΔΔCt^ method. Results were represented as means ± standard error of mean (SEM). The results were statistically analyzed by two-way ANOVA test followed by a posthoc unpaired t-test. For the majority of the sets (each time-course expression of each nematode strain), except for polyQ-YFP expression in muscles (Fig. 1a) and polyQ-CFP expression in pan-neuronal cells (Fig. 3a), the test revealed no interaction between the two parameter, polyQ-length (strain) and age. In all data sets there was a significant correlation between time and strain and the significance for each time point was assessed by means of unpaired t-test integrated in the GraphPad Prism 5 program. Differences with *p* < 0.05 were considered as significant.

### Immunoblotting analysis and filter-retardation assay

A defined number of synchronized individuals were collected in 10 μl of nuclease-free H_2_O and lysed in SDS sample buffer (313 mM Tris-HCl pH 6.8, 5% SDS, 0.5% Bromphenol blue, 50% glycerol, freshly added 5% β-mercaptoethanol). Samples were boiled for 15 min and resolved on a 12% SDS polyacrylamide gel. The polyQ variants were detected by immunoblotting using anti-GFP (1:2000; Roche) as a primary antibody for detection of YFP and CFP counterparts and visualized by chemiluminescence via a secondary goat-anti mouse antibody coupled with horseradish peroxidase (1:10000; BioRad). The quantification of the detergent-soluble Q-YFP or Q-CFP monomers was performed from the recorded tif-images of the immunoblots and represents the integrated pixel intensity of the corresponding band with subtracted intensity of the background. Note that the quantification for polyQ proteins can be only approximate because of the intrinsic length-dependent propensity to form SDS-insoluble aggregates which remain unresolved on the gel; also polyQ proteins with non-pathological polyQ lengths form small fractions of SDS-resistant aggregates^50^.

For filter-retardation assays^26^, a defined number of synchronized *C. elegans* individuals was picked in 50 μl of nuclease-free H_2_O and lysed in 500 μl lysis buffer (50 mM DTT, 2% SDS). Samples were boiled for 15 min and then loaded onto a cellulose acetate membrane with a pore size of 0.2 μm. SDS-insoluble aggregates were detected by immunoblotting using anti-GFP (1:2000; Roche) as a primary antibody for detection of YFP and CFP counterparts and visualized by chemiluminescence.

### Analysis of microarray data

We downloaded the data sets from the expression profiling in young and aged *C. elegans*^18^ and the IDs of the transcripts for which the authors detected significant changes between transcript levels in one-day and six-day old nematodes were extracted from the Ensembl Genome browser^51^. To identify tissue-specific groups genes were binned based on their GO terms^30^. For muscles we extracted 237 significantly changed genes and for pan-neuronal tissues 357. Both separately analyzed GO terms, GO:0005859 (muscle myosin complex) and GO: 0032809 (neuronal cell body), contain 14 and 13 genes with significantly changed mRNA expression pattern in aged animals, respectively.

## Additional Information

**How to cite this article**: Adamla, F. & Ignatova, Z. Somatic expression of *unc*-54 and *vha*-6 mRNAs declines but not pan-neuronal *rgef*-1 and *unc*-119 expression in aging *Caenorhabditis elegans*. *Sci. Rep.* **5**, 10692; doi: 10.1038/srep10692 (2015).

## Supplementary Material

Supporting Information
