# Supplementary material for: Somatic expression of unc-54 and vha-6 mRNAs declines but not pan-neuronal rgef-1 and unc-119 expression in aging Caenorhabditis elegans
Source: Sci Rep. 2015 Jun 2;5:10692. doi: 10.1038/srep10692 (PMC4649908; doi:10.1038/srep10692)
Supplement: Supporting Information [file srep10692-s1.pdf]

SUPPLEMENTARY INFORMATION

Somatic expression of *unc-54* and *vha-6* mRNAs declines but not pan-neuronal *rgef-1* and *unc-119* expression in aging *Caenorhabditis elegans*

Frauke Adamla[1] and Zoya Ignatova[1,2]

[1]Biochemistry, Institute of Biochemistry and Biology, University of Potsdam, Potsdam, Germany

[2]Biochemistry, Dept of Chemistry and Biochemistry, University of Hamburg, Hamburg, Germany

Table S1. Sequences of the primers used in qRT-PCR.

Gene (abbreviation)	Gene name	Sequence (5'→3')[a]
act-2 (first set)	Actin	f: CCCAATCCAAGAGAGGTATCCTT<br>r: GAAGCTGGTTGTAGAAAGTGTGATG
act-2 (second set)[b]	Actin	f: GCGCAAGTACTCCGTCTGGATCG<br>r: GGGTGTGAAAATCCGTAAGGCAGA
YFP/CFP	Yellow/cyan fluorescent protein	f: CGACCACTACCAGCAG<br>r: CTCCAGCAGGACCATG
vha-6 (first set)	Vacuolar H-ATPase	f: CAAGAGCAGAGATGCAAG<br>r: AGGATAAGTTCAACGAGGG
vha-6 (second set)	Vacuolar H-ATPase	f: GCGTTTCCCACACTGCTTCATAC<br>r: TCCAGAGAACGTCGGAAAGCTG
unc-54 (first set)	Heavy myosin chain	f: AGAAGCAGGTTGAGGAAGCTGAG<br>r: TTCAAGTTGGTGGGTGAGTTGC
unc-54 (second set)[c]	Heavy myosin chain	f: GAGAAGACCGAAGACATGTC<br>r: CAAATGTGGTGGCATTTCTGT
ama-1[d]	Amanitin resistance gene encoding for the large subunit of RNA polymerase II	f: CGGATGGAGGAGCATCGCCG<br>r: CAGCGGCTGGGGAAGTTGGC
unc-15[e]	Paramyosin	f: GGAGGATACTCAACGTCAGTTGC<br>r: CGGATAGCGTTGTCGAGAGCGG
unc-119	Required for proper development of the nervous system	f: CGAATGATTGAACGGCGGCACTTTTTCAAGG<br>r: GTCGTCCATGAGTTGTTGTGAAAGTTG

[a]f-forward primer; r – reverse primer; [b,d]The same primers were used as described in ([1]), [c]The same primers were used as described in ([2]), [e]The same primers were used as described in ([3])

References

1. Zhang, Y., Chen, D., Smith, M.A., Zhang, B. & Pan, X. Selection of reliable reference genes in Caenorhabditis elegans for analysis of nanotoxicity. *PLoS One* **7**, e31849 (2012).
2. van Oosten-Hawle, P., Porter, R.S. & Morimoto, R,I. Regulation of organismal proteostasis by transcellular chaperone signaling. *Cell* **153**, 1366-1378 (2013).
3. Ben-Zvi, A., Miller, E.A. & Morimoto, R.I. Collapse of proteostasis represents an early molecular event in Caenorhabditis elegans aging. *Proc. Natl. Acad. Sci. USA* **106**, 14914-14919 (2009).

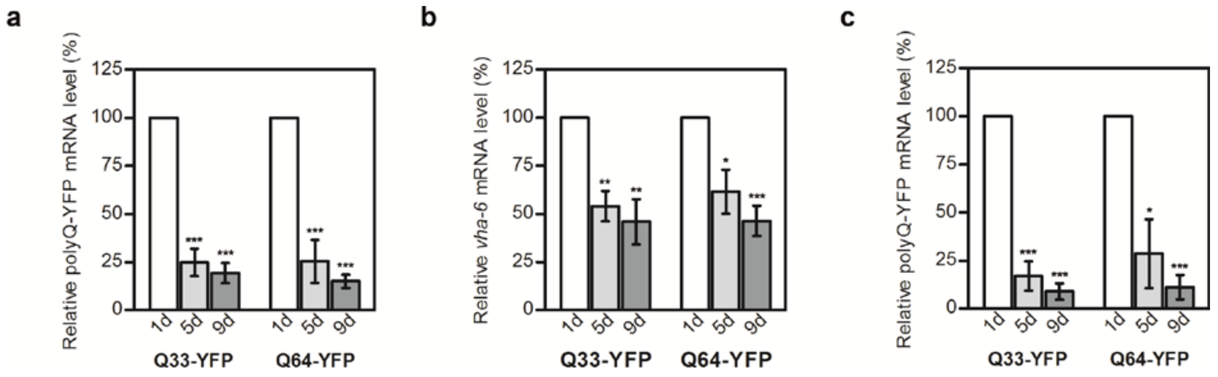

Figure S1. Quantification of polyCAG mRNA level in the intestine of aged *C. elegans* measured by qRT-PCR using a second set of *act-2* (**a**) or *vha-6* primers (**b**) (Supplementary Table S1), or normalized to a different housekeeping gene, *ama-1* (**c**). Data are presented as relative values normalized to the first day of adulthood ± SEM (n = 3-4, * $p < 0.05$, ** $p < 0.01$, *** $p < 0.001$.

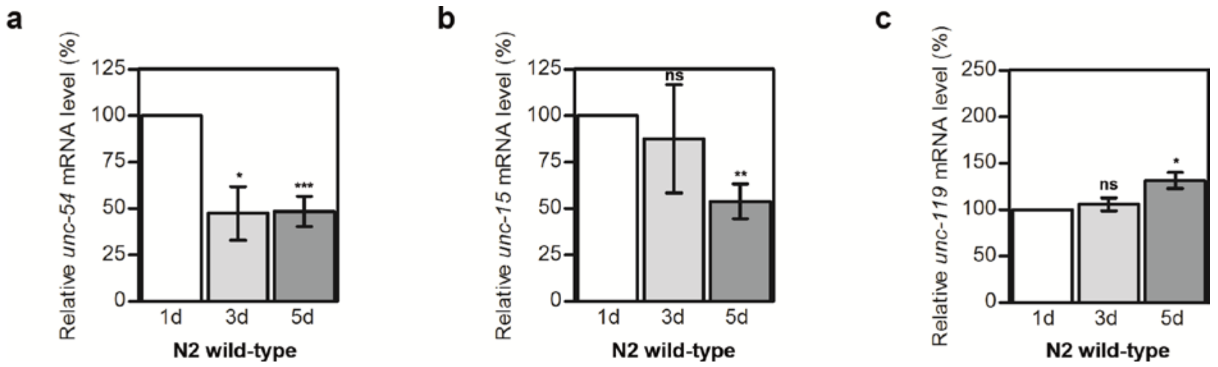

Figure S2. Quantification of *unc-54* (**a**) and *unc-15* (**b**) mRNA levels in the muscle cells of aged N2 wild-type animals measured by qRT-PCR using a second primer set for the *unc-54* gene (**a**). *act-2* was used as a reference housekeeping gene for normalization. (**c**) Quantification of *unc-119* mRNA in pan-neuronal tissues normalized to the expression of the housekeeping *ama-1* gene. Data are presented as relative values normalized to the first day of adulthood ± SEM (n = 3-4, *$p < 0.05$, ** $p < 0.01$, *** $p < 0.001$, ns, non-significant).
